# The effect of omega-3 fatty acid supplementation on clinical and biochemical parameters of critically ill patients with COVID-19: a randomized clinical trial

**DOI:** 10.1186/s12967-021-02795-5

**Published:** 2021-03-29

**Authors:** Saeid Doaei, Somayeh Gholami, Samira Rastgoo, Maryam Gholamalizadeh, Fatemeh Bourbour, Seyedeh Elaheh Bagheri, Forough Samipoor, Mohammad Esmail Akbari, Mahdi Shadnoush, Fereshteh Ghorat, Seyed Alireza Mosavi Jarrahi, Narjes Ashouri Mirsadeghi, Azadeh Hajipour, Parvin Joola, Alireza Moslem, Mark O. Goodarzi

**Affiliations:** 1 Research Center of Health and Environment, School of Health, Guilan University of Medical Sciences, Rasht, Iran; 2 Cancer Research Center, Shahid Beheshti University of Medical Sciences, Tehran, Iran; 3 Razi Hospital, Guilan University of Medical Sciences, Rasht, Iran; 4 National Nutrition and Food Technology Research Institute, Faculty of Nutrition Sciences and Food Technology, Shahid Beheshti University of Medical Sciences, Tehran, Iran; 5 Student Research Committee, Cancer Research Center, Shahid Beheshti University of Medical Sciences, Tehran, Iran; 6 School of Paramedicine, Guilan University of Medical Sciences, Langroud, Iran; 7 Department of Clinical Nutrition, Faculty of Nutrition Sciences and Food Technology, National Nutrition and Food Technology Research Institute, Shahid Beheshti University of Medical Sciences, Tehran, Iran; 8 Traditional and Complementary Medicine Research Center, Sabzevar University of Medical Sciences, Sabzevar, Iran; 9 School of Health, Qazvin University of Medical Sciences, Qazvin, Iran; 10 Department of Non-Communicable Disease, Deputy of Health Services, Dezful University of Medical Sciences, Dezful, Iran; 11 Iranian Research Center on Healthy Aging, Sabzevar University of Medical Sciences, Sabzevar, Iran; 12 Division of Endocrinology, Diabetes and Metabolism, Department of Medicine, Cedars-Sinai Medical Center, Los Angeles, CA USA

**Keywords:** Omega-3 fatty acids, Coronavirus, Kidney function, Respiratory function

## Abstract

**Background:**

Omega-3 polyunsaturated fatty acids (n3-PUFAs) may exert beneficial effects on the immune system of patients with viral infections. This paper aimed to examine the effect of n3-PUFA supplementation on inflammatory and biochemical markers in critically ill patients with COVID-19.

**Methods:**

A double-blind, randomized clinical trial study was conducted on 128 critically ill patients infected with COVID-19 who were randomly assigned to the intervention (fortified formula with n3-PUFA) (n = 42) and control (n = 86) groups. Data on 1 month survival rate, blood glucose, sodium (Na), potassium (K), blood urea nitrogen (BUN), creatinine (Cr), albumin, hematocrit (HCT), calcium (Ca), phosphorus (P), mean arterial pressure (MAP), O_2_ saturation (O_2_sat), arterial pH, partial pressure of oxygen (PO_2_), partial pressure of carbon dioxide (PCO_2_), bicarbonate (HCO_3_), base excess (Be), white blood cells (WBCs), Glasgow Coma Scale (GCS), hemoglobin (Hb), platelet (Plt), and the partial thromboplastin time (PTT) were collected at baseline and after 14 days of the intervention.

**Results:**

The intervention group had significantly higher 1-month survival rate and higher levels of arterial pH, HCO_3_, and Be and lower levels of BUN, Cr, and K compared with the control group after intervention (all P < 0.05). There were no significant differences between blood glucose, Na, HCT, Ca, P, MAP, O2sat, PO_2_, PCO_2_, WBCs, GCS, Hb, Plt, PTT, and albumin between two groups.

**Conclusion:**

Omega-3 supplementation improved the levels of several parameters of respiratory and renal function in critically ill patients with COVID-19. Further clinical studies are warranted.

*Trial registry*

Name of the registry: This study was registered in the Iranian Registry of Clinical Trials (IRCT); Trial registration number: IRCT20151226025699N3; Date of registration: 2020.5.20; URL of trial registry record: https://en.irct.ir/trial/48213

## Introduction

In recent years, coronavirus infections have been a significant source of morbidity and mortality around the world [1]. Novel coronavirus 2019 (nCoV-2019), now known as acute respiratory syndrome coronavirus 2 (SARS-CoV-2), is responsible for the pandemic of coronavirus disease COVID-19 [2]. This infection usually causes a defined pattern of metabolic and clinical changes in affected patients [3]. Leukocytopenia, lymphopenia, and elevated C-reactive protein (CRP) can occur in the primary form of COVID-19. With disease progression, increasing levels of leukocytes, creatine kinase, and creatinine may also occur [4].

Host nutritional status has an important role in defense against viral infections [5]. Numerous studies have reported that malnourished humans are more susceptible to a wide variety of infections [6]. Indeed, proper nutrition can preserve the immune system and improve its function [7]. Nutritional deficiencies influence both immune response and viral pathogenic functions [5, 8]. Moreover, they may lead to oxidative stress in the host which can alter the genome of the virus, so that a normally benign or mildly pathogenic virus can convert to a highly virulent pathogen [6].

Omega-3 polyunsaturated fatty acids (n3-PUFAs) are important mediators of inflammation and acquired immune responses and can amplify anti-inflammatory responses [9]. Recent studies have been shown that n3-PUFAs including eicosapentaenoic acid (EPA), docosahexaenoic acid (DHA), and α-linoleic acid (ALA) can increase the stability of the cell membrane, regulate immune function, block hyper inflammatory reactions, and reduce the incidence of systemic inflammatory response syndrome (SIRS), multiple organ dysfunction syndrome (MODS), and complications of infection [10].

Chen et al. reported a lower mortality rate in septic patients who received total parenteral nutrition containing 100 ml (ml) n3-PUFAs daily for 4 weeks. Also, the treatment group had a higher ratio of T helper to inducer lymphocytes and CD4 to CD8 lymphocytes in comparison with the control group during the first 7 days of follow-up [11]. Pluta et al. found that patients with chronic kidney disease, who daily received one capsule of omega 3 including 1000 mg n3-PUFAs containing 330 mg of EPA, 220 mg of DHA, and 100 mg of other acids such as alpha-linolenic acid (ALA) had lower urinary excretion of monocyte chemoattractant protein-1 (MCP-1) after 6 months of follow-up. However, white blood cell (WBC) count and serum levels of CRP and MCP-1 did not change significantly [12]. Soleimani et al. reported that patients with diabetic nephropathy, who received 1 g omega-3 acids daily had higher circulating nitric oxide (NO) after 12 weeks of follow-up with no change in serum concentrations of high sensitivity (hs)-CRP or total antioxidant capacity (TAC) [13]. Coghill et al. demonstrated that human immunodeficiency virus (HIV) and human herpesvirus-8 (HHV-8) co-infected patients, who received 3 gr of omega-3 fatty acids daily (each pill contained about 230 mg EPA and 150 mg DHA), had lower serum concentrations of the inflammatory cytokine interleukin-6 (IL-6) and higher CD8 + counts at 12 weeks [14]. Omar et al. in a recent study found that end-stage renal disease patients on chronic hemodialysis, who daily received two grams of omega-3 (containing 360 mg EPA and 240 mg DHA) had lower serum levels of IL-6 and CRP at 3 months [15]. On the other hand, Rice et al. conducted a randomized, double-blind, placebo-controlled, multicenter trial and reported that the n-3 supplementation did not improve the primary end point of ventilator-free days or other clinical outcomes in patients with acute lung injury and may be harmful. Patients receiving the n-3 supplement had fewer ventilator-free days, intensive care unit–free days, and nonpulmonary organ failure–free days [16]

Given suggestive evidence of the beneficial effects of n3-PUFAs on the immune system and the contradictory results of recent studies concerning the effects of omega-3 fatty acids in patients with viral infections, we aimed to examine the effect of omega-3 supplementation on inflammatory and biochemical markers in critically ill patients with COVID-19.

## Methods

### Study design and population

This study was a double-blind, randomized clinical trial study carried out from May to July 2020 in critically ill patients infected with COVID-19 in Razi Hospital, Rasht, Iran. The inclusion criteria were the age between 35 and 85 years, diagnosis of COVID-19 confirmed by a positive RT-PCR nasopharyngeal swab, as well as symptoms such as severe pneumonia, fever, fatigue, dry cough, respiratory distress, and indicated for enteral nutrition. One hundred thirty five patients were assessed for eligibility and 128 patients met the inclusion criteria. Sample size was calculated using α = 0.95, β = 20%, ratio of unexposed to exposed of 2:1, and power of 0.8. An unequal randomization ratio (2:1) was used because of a fixed and limited budget for this research project. Therefore, more participants were randomized to the cheaper arm in order to facilitate greater overall recruitment in the face of a possibly high drop-out rate. The exclusion criteria included history of cardiovascular and/or lung diseases (n = 4), and diagnosis of malignant tumors (n = 1) because of their effects on current medical treatment and diet, consumption of omega-3 fatty acids during the prior 3 months before the study (n = 1), history of hypersensitivity reactions to fish or its products (n = 1), did not complete the study because of death (n = 14) or no indication for enteral feeding any more (n = 13). The final analysis was performed on 101 critically ill patients infected with COVID-19 who were assigned to the intervention (n = 28) and control (n = 73) groups.

### Interventions

This study was done in form of a double-blind trial. Though no placebo was used, the patients were not aware of their feeding contents, and the patients and researchers were not aware of the arms of the study. Finally, the results were analyzed by a person outside of the treatment team. The allocation to the groups was done through web-based randomization using https://www.randomizer.org. Sealed non-transparent envelopes with randomized sequences were used to hide the allocation. All participants received high protein formula as 30 kcal/kg/d through enteral feeding. The intervention group received one capsule of 1000 mg omega-3 daily (Vita Pharmed, Switzerland) containing 400 mg EPAs and 200 mg DHAs for 14 days through adding the supplement to their enteral formula. Omega-3 fatty acids fortified formula was administered to the case group by a nurse, who was not a member of the research team, for 2 weeks after the first 24 h of hospitalization in ICU. The omega-3 capsules were pierced with a syringe, then its contents were squeezed out into a prepared enteral formula, with thorough mixing. The control group received nutritional support including the isocaloric-isovolemic formula using the same route, except for the intervention.

### Data collection

After collecting the written consent forms, the required information was collected using a pre-determined checklist including the following sub-scales:

### Anthropometric and medical history

Data on age, weight, height, previous illnesses, medications, blood pressure, serum lipids, random blood glucose, and respiratory status was collected using medical records.

### Dietary intake

Data on nutritional intake including caloric intake, type of formula, and dietary supplements were assessed using documents recorded in ICU sheets and patient logs.

### Biochemical and pathological indices

Arterial blood gas (ABG) parameters [i.e., O_2_ saturation, arterial pH, partial pressure of oxygen (PO_2_), partial pressure of carbon dioxide (PCO_2_), bicarbonate (HCO_3_), and base excess (Be)], kidney function parameters [i.e., blood urea nitrogen (BUN), creatinine (Cr), and urine volume], as well as blood glucose, mean arterial pressure, white cell blood count (including neutrophil, lymphocyte, monocyte), the Acute Physiology and Chronic Health Evaluation II (APACHE II), Glasgow Coma Scale (GCS), hemoglobin (Hb), platelet (Plt), the partial thromboplastin time (PTT), serum electrolytes [i.e. sodium (Na), potassium (K), calcium, phosphorus (P)], albumin, and hematocrit (Hct) were measured at baseline and after 14 days of the intervention. These measurements were routinely performed in the hospital using standard kits and the required data were gathered from the lab test section of the ICU sheets.

### Statistical analyses

Quantitative data were presented as the mean ± standard deviation and qualitative data were presented as the percentage and number to describe the status of demographic, social, and anthropometric measurements of the participants. The Kolmogorov–Smirnov test was used to verify the normal distribution. Independent *T*-test and chi-square test were used to compare results between groups at baseline. The core research question was whether the difference between groups of interest changed from the basal values to the values after treatment. The general linear model repeated measure was used to identify the group-by-time interaction effect of omega-3 supplementation on inflammatory and biochemical markers, comparing the values in intervention and control groups. Confounding factors including age, sex, body mass index (BMI), dietary intake, smoking, presence of background diseases such as diabetes and hypertension, and medication and supplements were adjusted for. Statistical analyses were performed using the SPPS software (version 20). P values less than 0.05 were considered statistically significant.

## Results

### Patient characteristics

**Fig. 1:**
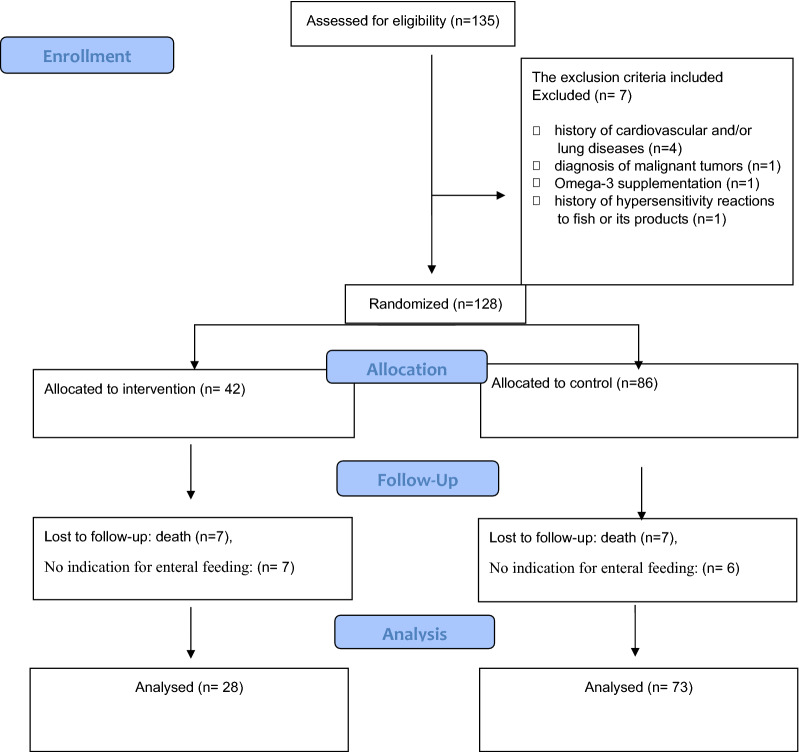
Study flowchart

A total of 135 eligible patients were included in this study and randomization was done 1:2. Of these, seven patients were excluded based on the exclusion criteria (Fig. 1). In addition, another seven patients in the treatment group and twenty controls were excluded from analysis after randomization, because they died during the intervention period. Finally, 101 patients were included in the analysis, of which 28 were assigned to the treatment group and 73 were assigned to the control group. All baseline data in Table 1 were normally distributed. The groups were not significantly different in demographic or clinical characteristics at baseline, except for the lymphocyte count, which was lower in the intervention group (11.59 vs 13.26, P = 0.01).

**Table 1:** Baseline demographic and clinical data of the study groups

	Interventions (n = 28)	Controls (n = 73)	P
Age y	66 (14.58)	64 (14.25)	0.12
Males n (%)	15 (53.60)	45 (61.60)	0.46
Weight kg	77.39 (21.12)	75.09 (7.26)	0.58
Height meter	1.67 (7.28)	1.66 (6.42)	0.39
BMI kg/m^2^	27.68 (7.54)	27.39 (3.15)	0.85
APACHE II	15.54 (1.73)	15.42 (1.92)	0.78
Blood group n (%)			
A	13 (50)	24 (34.3)	0.14
B	3 (11.5)	16 (22.9)	
AB	1 (3.8)	0	
O	9 (34.6)	30 (42.9)	
Addiction n (%)			
Yes	1 (3.6)	6 (8.6)	0.39
No	27 (96.4)	64 (91.4)	
Tobacco n (%)			
Yes	2 (7.1)	5 (7.1)	1
No	26 (92.9)	65 (92.9)	
Underlying disease n (%)			
Yes	14 (50)	28 (40)	0.57
No	14 (50)	41 (60.2)	
1 month survival rate	5 (21)	2 (3)	0.005
Blood glucose mg/mL	148.98 (10.00)	152.41 (8.71)	0.15
Na mEq/L	138.48 (3.47)	139.32 (1.74)	0.11
K mEq/L	4.03 (0.1)	4.00 (0.1)	0.51
BUN mg/mL	37.06 (4.14)	35.17 (4.10)	0.96
Cr mg/mL	1.33 (0.25)	1.29 (0.24)	0.76
Alb g/dL	3.02 (0.14)	3.35 (0.39)	0.44
Hct %	31.88 (0.61)	29.65 (0.66)	0.59
Ca mg/dL	7.96 (0.22)	7.65 (0.12)	0.80
P mg/dL	3.30 (0.21)	3.33 (0.19)	0.87
MAP mmHg	74.23 (1.64)	74.42 (1.74)	0.97
O2 sat. %	84.47 (1.93)	77.79 (1.80)	0.16
Arterial pH	7.27 (0.01)	7.30 (0.01)	0.15
PO_2_ mmHg	68.93 (3.72)	67.72 (3.97)	0.53
PCO_2_ mmHg	45.20 (2.41)	43.53 (2.03)	0.06
HCO_3_ mEq/L	21.11 (1.13)	22.00 (1.19)	0.91
Be mEq/L	− 4.97 (1.04)	− 3.59 (1.05)	0.11
WBC 10^6^/L	14.26 (1.12)	20.47 (3.83)	0.40
Neutrophil 10^6^/L	86.54 (0.74)	86.76 (0.94)	0.22
Lymphocyte 10^6^/L	11.59 (0.55)	13.26 (0.81)	0.01
Monocyte 10^6^/L	2.12 (0.26)	1.67 (0.28)	0.11
GCS	8.37 (0.22)	7.90 (0.20)	0.09
Hb g/dL	10.32 (0.28)	9.50 (0.26)	0.07
Plt	248.69 (16.27)	187.91 (16.18)	0.07
Ptt second	38.90 (2.20)	43.07 (2.52)	0.10
Urine volume mL/day	1572.60 (201.15)	1803.44 (180.29)	0.05
Number of dialysis days	0.26 (0.66)	0.19 (0.72)	0.67
Number of insulin injection days	0.26 (0.71)	0.07 (0.50)	0.23

Data are mean (SD) for quantitative variables and number (%) for categorical variables

*sBMI*  body mass index, *APACHE* II  acute physiologic assessment and chronic health evaluation II, *Na*  sodium, *K*  potassium, *BUN*  blood urea nitrogen, *Cr*  creatinine, *Alb*  albumin, *HCT*  hematocrit, *Ca*  calcium, *P* phosphorus, *MAP*  mean arterial pressure, *O2* *sat*.  oxygen saturation, *pH*  potential hydrogen, *PO*_*2*_  partial pressure of oxygen, *PCO2*  partial pressure of carbon dioxide, *HCO*_*3*_  bicarbonate, *Be*  base excess, *WBC*  white blood cell, *GCS*  glasgow coma score, *Hb*  hemoglobin, *Plt*  platelet, *Ptt*  partial thromboplastin time (test)

### Effects of omega-3 supplementation on 1-month survival rate

The intervention group had significantly higher 1-month survival rate compared with the control group (21% vs 3%, P = 0.003). About 21% (n = 6) of the participants in the intervention group and only about 3% (n = 2) of the participants in the control group survived at least for 1 month after the beginning of the study.

### Effects of omega-3 supplementation on kidney function

As summarized in Table 2, levels of BUN (35.17 vs 43.19, F = 4.76, P = 0.03) and Cr (1.29 vs 1.68, F = 5.90, P = 0.02) were significantly lower and the amount of urine excreted (2101 vs 1877.02, F = 12.26, p = 0.01) was significantly higher in the intervention group compared with the control group.

**Table 2:** Comparison of clinical and biochemical parameters between the intervention group and control group after the intervention

Factors	Intervention group mean (SE)	Control group mean (SE)	Group time^a^	
	After	After	F	P
Blood sugar (mg/mL)	152.41 (8.71)	142.27 (5.35)	0.16	0.69
Na (mEq/L)	139.32 (1.74)	140.99 (1.08)	1.25	0.27
K (mEq/L)	4.00 (0.1)	4.14 (0.06)	10.15	0.01
BUN (mg/mL)	35.17 (4.10)	43.19 (2.50)	4.76	0.03
Cr (mg/mL)	1.29 (0.24)	1.68 (0.15)	5.90	0.02
Alb (g/dL)	3.35 (0.39)	2.88 (0.26)	0.59	0.45
Hct (%)	29.65 (0.66)	29.84 (0.43)	0.08	0.77
Ca (mg/dL)	7.65 (0.12)	7.88 (0.72)	0.27	0.61
P (mg/dL)	3.33 (0.19)	3.12 (0.12)	0.80	0.78
MAP (mmHg)	74.42 (1.74)	69.72 (1.06)	3.25	0.08
O_2_ sat. (%)	80.73 (1.80)	75.50 (1.11)	0.13	0.72
Arterial pH	7.30 (0.01)	7.26 (0.01)	19.11	0.01
PO_2_ (mmHg)	67.72 (3.97)	61.40 (2.58)	0.00	0.96
PCO_2_ (mmHg)	43.53 (2.03)	44.26 (1.25)	1.55	0.22
HCO_3_ (mEq/L)	22.00 (1.19)	18.17 (0.73)	10.83	0.01
Be (mEq/L)	− 3.59 (1.05)	− 7.09 (0.65)	23.01	0.01
WBC (10^6^/L)	20.47 (3.83)	13.57 (2.35)	0.36	0.55
Neutrophil (10^6^/L)	86.76 (0.94)	87.29 (0.85)	2.00	0.17
Lymphocyte (10^6^/L)	11.59 (0.81)	11.80 (0.73)	4.08	0.05
Monocyte (10^6^/L)	1.67 (0.28)	2.16 (0.42)	0.03	0.87
GCS	7.90 (0.20)	7.49 (0.12)	6.07	0.05
Hb (g/dL)	9.50 (0.26)	9.68 (0.16)	1.60	0.21
Plt	187.91 (16.18)	162.49 (9.69)	1.11	0.30
Ptt (second)	43.07 (2.52)	44.14 (1.55)	1.81	0.18
Urine volume (mL)	2101 (884)	1877.02 (917)	12.26	0.01

*sBMI*  body mass index, *APACHE* II  acute physiologic assessment and chronic health evaluation II, *Na*  sodium, *K*  potassium, *BUN*  blood urea nitrogen, *Cr*  creatinine, *Alb*  albumin, *HCT*  hematocrit, *Ca*  calcium, *P* phosphorus, *MAP*  mean arterial pressure, *O2* *sat*.  oxygen saturation, *pH*  potential hydrogen, *PO*_*2*_  partial pressure of oxygen, *PCO2*  partial pressure of carbon dioxide, *HCO*_*3*_  bicarbonate, *Be*  base excess, *WBC*  white blood cell, *GCS*  glasgow coma score, *Hb*  hemoglobin, *Plt*  platelet, *Ptt*  partial thromboplastin time (test)

^a^Repeated measure ANOVA

### Effects of omega-3 supplementation on arterial blood gas (ABG) parameters

After 14 days, the levels of arterial pH (7.30 vs 7.26, F = 19.11, P = 0.01), HCO_3_ (22.00 vs 18.17, F = 10.83, P = 0.01), and Be (−4.97 vs −3.59, F = 23.01, P = 0.01) were significantly higher in the intervention group compared with the control group. However, there were no significant differences among PO_2_ and PCO_2_ between two groups (Table 2).

### Effects of omega-3 supplementation on the Glasgow coma scale (GCS)

On admission to the study, the mean GCS was 8.37 in the intervention group and 7.90 in the control group (P > 0.05). As shown in Table 2, it was significantly lower after 14 days of the study in both groups (7.90 vs 7.49, F = 6.07, P = 0.05). No significant difference was found in APACHE II score between the groups (15.54 ± 1.73 vs 15.42 ± 1.92., P = 0.78).

### Effects of omega-3 supplementation on serum electrolytes

As shown in Table 2, the level of K was significantly reduced in the intervention group compared to the control group (4.00 vs 4.14, F = 10.15, P = 0.01) after 14 days. No significant differences were found between the levels of serum electrolytes including Na, Ca, and P in between two groups after intervention.

### Effects of omega-3 supplementation on blood clotting function and cell blood count (CBC)

The lymphocyte count increased in the omega-3 group compared to the control group, though this effect was marginally significant (11.59 vs 11.80, F = 4.08, P = 0.05). There were no significant differences in levels of PTT, hematocrit, neutrophil, monocyte, hemoglobin, and Plt between the two groups (Table 2).

### Effects of omega-3 supplementation on the other blood factors

As shown in Table 2, no significant differences were observed in the other factors including blood glucose, albumin, MAP, and O_2_ sat between two groups after intervention.

## Discussion

To our knowledge, this is the first randomized clinical trial assessing the effect of omega-3 fatty acid supplementation in ICU patients with COVID-19. We found that administration of omega-3 PUFA significantly improved arterial PH, HCO_3_, and Be. This trial also supports our hypothesis that omega-3 supplementation can improve the level of indicators of kidney function including BUN, Cr, K, and urine volume. The results have indicated that omega-3 supplementation may increase the lymphocyte count and GCS. However, these increases were not statistically significant. In our study, omega-3 supplementation had a positive effect on 1-month survival rate of the critically ill patients with COVID-19.

Many severely or critically ill COVID-19 patients have been reported to develop severe metabolic and respiratory acidosis [17, 18], indicating possible microcirculation dysfunction [18]. This study identified that omega-3 supplementation improved arterial PH, HCO_3_, and Be, which may be related to the effect of omega-3 supplementation on microcirculatory function. Several studies [19–21] have reported that omega-3 supplementation improved endothelial function and microcirculation. Vasil'ev et al. reported that therapy with omega-3 PUFAs increased tissue hemoperfusion, capillary blood flow, and tissue blood flow and generally improved microcirculation. Trevor et al. studied the effects of DHA and EPA supplementation on vascular reactivity and microcirculation. They found that DHA significantly enhanced vasodilator mechanisms, attenuated constrictor responses, and improved endothelium reactivity and microcirculation [22]

It has been reported that renal abnormalities occurred in the majority of patients with COVID-19 [18, 23–25], and microemboli in renal vessels as a consequence of the prothrombotic state results in kidney damage. Furthermore, hypertension induced by pro-inflammatory status, increasing IFN-γ, IL-6, and IL-17 expression and CD8 + T cells in the kidney, angiotensin II-induced hypertension, and the endothelial dysfunction caused by the innate immune response seems to be involved in the kidney injury [26]. Renal involvement in COVID-19 was reported to be associated with higher mortality [18, 23–25]. Hence, it is crucial to identify adjuvant and protective therapies that improve kidney function in patients with severe COVID-19. The present study found that the administration of omega-3 PUFAs significantly decreased BUN, Cr, and K and increased urine volume in ICU patients infected with COVID-19. These findings suggest that omega-3 supplementation may be protective against progression to renal impairment, consistent with observations from animal models that showed that omega-3 PUFA supplementation reduces the progression of renal disease [27–31]. Moreover, studies in human subjects suggested that a higher dietary intake of PUFAs may improve creatinine clearance [32] and also may have a role in maintaining healthy kidney function [33]. In contrast, some studies have reported that omega-3 treatment did not protect renal function. Higashihara et al. assessed the effects of omega-3 PUFAs on kidney function in 41 patients with stages two or three chronic kidney disease (CKD); omega-3 supplementation did not alter kidney function, assessed using 24-h urine creatinine clearance. Another study demonstrated that omega-3 PUFA pre-treatment did not protect against renal function deterioration or ischemia-induced renal inflammation; however, tubular transport was improved [30]. Previous studies that have evaluated omega-3 PUFAs in the treatment of kidney disease have yielded contradictory results, which may reflect differences in study duration, dosage of omega-3, route of administration, and type of omega-3 fatty acids.

Omega-3 PUFAs can change the lipid composition and cell function of lymphocytes and affect cellular immune function [10, 34, 35]. There is no consensus regarding the efficacy of omega-3 PUFAs on lymphocyte count. A meta-analysis of randomized control trials found that the value of postoperative lymphocyte count increased in response to omega-3 PUFAs supplementation [10]. Consistent with this finding, the results of the present study identified a marginal increase in lymphocyte count after omega-3 PUFAs supplementation.

The results of the current study identified a significant difference in 1-month survival rate between two groups. Supplementation with omega-3 fatty acids may have immune-modulating [36–38] and organ-protective effects [39, 40]. Previous studies have reported that higher plasma levels of omega-3 PUFAs are associated with survival [41–43]. Moreover, a recent systematic review found that omega-3 supplements can improve survival in critically ill patients [44]. However, another recent review article concluded that there is weak evidence for beneficial effects of omega-3 supplementation on ICU or hospitalized survival in critically ill patients [45]. Heller et al. have reported that the effects and effect sizes of omega-3 on clinical outcome is dependent on the disease [46].

Our study had some limitations. Although the sample size was calculated with an acceptable power for the study, the results need to be confirmed in larger studies. Moreover, only one dosage of omega-3 was used, which makes it difficult to discuss the dose–response efficacy of the supplement. It is possible that we may have seen more benefit with a higher dose. Another limitation of this study was the short duration of the study. Furthermore, prognostic biochemical markers in COVID-19 including the inflammatory cytokines C-reactive protein (CRP), interleukin-6, and interleukin-10, were not measured due to limited resources. Another limitation of this study was that there was no access to an omega-3 fatty acid enriched formula, and therefore we had to add it manually.

## Conclusion

In conclusion, this randomized, double-blind, clinical trial has shown that omega-3 supplementation has promising effects on acidosis and renal function and possibly can improve clinical outcomes of patients infected with COVID-19. Further clinical studies with different dosages of n-3 PUFAs, larger sample sizes, and longer duration are warranted.

## Data Availability

All data will be made available upon request.

## Abbreviations

n3-PUFAs: Omega-3 polyunsaturated fatty acids
Na: Sodium
K: Potassium
BUN: Blood urea nitrogen
Cr: Creatinine
HCT: Hematocrit
Ca: Calcium
P: Phosphorus
MAP: Mean arterial pressure
O2 sat: O_2_ saturation
PCO_2_: Partial pressure of oxygen
WBC: White blood cells
